# USP32 facilitates non-small cell lung cancer progression via deubiquitinating BAG3 and activating RAF-MEK-ERK signaling pathway

**DOI:** 10.1038/s41389-024-00528-z

**Published:** 2024-07-19

**Authors:** Shuang Li, Lina Yang, Xiaoyan Ding, Hongxiao Sun, Xiaolei Dong, Fanghao Yang, Mengjun Wang, Huhu Zhang, Ya Li, Bing Li, Chunyan Liu

**Affiliations:** 1 Department of Genetics and Cell Biology, School of Basic Medicine, Qingdao University, 266071 Qingdao, China; 2 School of Basic Medicine, Institute of Stem Cell and Regenerative Medicine, Qingdao University, 266071 Qingdao, China; 3 Heart Center, Women and Children’s Hospital, Qingdao University, 6 Tongfu Road, 266034 Qingdao, China; 4 Department of Dermatology, The Affiliated Haici Hospital of Qingdao University, 266000 Qingdao, China

**Keywords:** Ubiquitylation

## Abstract

The regulatory significance of ubiquitin-specific peptidase 32 (USP32) in tumor is significant, nevertheless, the biological roles and regulatory mechanisms of USP32 in non-small cell lung cancer (NSCLC) remain unclear. According to our research, USP32 was strongly expressed in NSCLC cell lines and tissues and was linked to a bad prognosis for NSCLC patients. Interference with USP32 resulted in a significant inhibition of NSCLC cell proliferation, migration potential, and EMT development; on the other hand, USP32 overexpression had the opposite effect. To further elucidate the mechanism of action of USP32 in NSCLC, we screened H1299 cells for interacting proteins and found that USP32 interacts with BAG3 (Bcl2-associated athanogene 3) and deubiquitinates and stabilizes BAG3 in a deubiquitinating activity-dependent manner. Functionally, restoration of BAG3 expression abrogated the antitumor effects of USP32 silencing. Furthermore, USP32 increased the phosphorylation level of the RAF/MEK/ERK signaling pathway in NSCLC cells by stabilizing BAG3. In summary, these findings imply that USP32 is critical to the development of NSCLC and could offer a theoretical framework for the clinical diagnosis and management of NSCLC patients in the future.

## Introduction

Lung cancer is the leading cause of cancer-related fatalities worldwide, for approximately 25% of the deaths related to cancer [1, 2]. The primary histologic subtype of lung cancer is non-small cell lung cancer (NSCLC), which encompasses a number of different forms, including lung adenocarcinoma (LUAD), lung squamous cell carcinoma (LUSC), and large cell carcinoma (LCC) [3]. The majority of patients are diagnosed with locally advanced disease or metastases to distant locations, because there is a dearth of an early diagnostic platform and patients typically present with late onset symptoms [4]. A number of novel therapies, including screening, minimally invasive procedures, radiation chemotherapy, etc., have advanced significantly in recent years thanks to the advancement of genetic analysis and molecular testing techniques [5]. Consequently, NSCLC patient survival rates have increased. Additionally, NSCLC patients can receive immunotherapy and molecular targeted therapy as a form of selective treatment because tumor biomarkers are continually being discovered and studied [6]. However, the microenvironment of NSCLC is subject to diverse changes due to genetic alterations and tissue-specific responses [7], so the analysis of genes and mechanistic studies is the discovery of new immunotherapeutic targets for NSCLC.

The association between malignant tumors and ubiquitin-proteasome pathway dysregulation has gained significant attention in recent years, and numerous studies have demonstrated that addressing this system may be a highly effective antitumor treatment approach [8, 9]. Similar to other post-translational modifications, ubiquitination is reversible and can be undone by a broad class of proteases known as deubiquitinating enzymes (DUBs) [10]. The DUB family member ubiquitin-specific peptidase 32 (USP32) has several structural domains that are involved in its function. It also has the most significant structural domain among USP members, the USP catalytic domain [11, 12]. USP6 and PoUSP32, as two homologs of USP32, share functional enzymatic structural domains and perform similar physiological functions as USP32 [13, 14]. USP32 is broadly distributed in human tissues, with the testis being the most highly expressed organ [11]. Research has demonstrated that USP32 is expressed in the cytoplasm as well as the cell membrane. It is also one of the few DUBs connected to the endocytosis pathway that can regulate the function of a number of factors [15, 16]. In the meantime, USP32 participates in physiological processes like cell cycle, invasion, migration, multiplication of cells, and repair of DNA damage [11]. Aberrant expression of USP32 triggers certain diseases such as Parkinson’s, Fragile X Syndrome, Chronic Kidney Disease and Cancer [11, 17–19]. Recent research has demonstrated the high expression of USP32 in a range of cancers [20–26] and its role in the initiation and progression of cancer. For example, USP32 influences the invasion, migration, and proliferation of small cell lung cancer [21]; USP32 affects the development of epithelial ovarian cancer [20], glioblastoma [22], and gastric cancer [23] through the regulation of related proteins. USP32 interacts with Rab35 due to the deubiquitinating enzyme activity of USP32, therefore promoting the mesenchymal malignancies in the gastrointestinal tract to acquire imatinib resistance [27]. Nevertheless, it is unknown what precise biological role USP32 plays and how it manifests in NSCLC.

Having 575 amino acids and serving several purposes, Bcl2-associated athanogene (BAG) 3 is a multifunctional co-chaperone and anti-apoptotic protein, which belongs to the BAG family [28, 29]. BAG3 is commonly expressed in human tissues, but it is most heavily expressed in the central nervous system, skeletal muscle, and heart. It is also highly expressed in several malignancies [28]. BAG3 controls intracellular protein stability and life processes by participating in the proteasomal and autophagic protein degradation pathways [30, 31]. Many neurological illnesses, cardiomyopathies, and malignancies have all been linked to abnormalities in BAG3 [32]. In cancer specifically, BAG3 has been demonstrated to enhance the growth and migration of a range of tumor cells [31, 33]. In the clinic, researchers have used BAG3-H2L4 antibodies for targeted therapy in pancreatic cancer [34]. Research has demonstrated that BAG3 is significantly expressed in non-small cell lung cancer (NSCLC) and that it can increase NSCLC cell survival via controlling associated proteins [35]. Therefore, revealing the regulatory mechanism of BAG3 will provide new perspectives for understanding the pathogenesis of NSCLC and new therapeutic strategies against NSCLC.

According to the current investigation, there was an overexpression of USP32 in NSCLC, and this overexpression was linked to a poor prognosis. Mechanistic investigations revealed that BAG3 is a substrate for USP32, and that USP32 directly interacts with BAG3 to promote NSCLC carcinogenesis through blocking BAG3 degradation. In addition, it was discovered that USP32 actives the RAF/MEK/ERK signaling pathway and stabilizes BAG3 expression to support the development of NSCLC. Overall, our work reveals USP32 as a potential therapeutic target for patients with non-small cell lung cancer and complements the deubiquitination regulatory network of BAG3.

## Materials and methods

### Immunohistochemistry (IHC) and analysis

The Affiliated Hospital of Qingdao University provided thirty-four pairs of NSCLC paraneoplastic and noncancerous tissue specimens undergoing surgical tumor excision. Patients gave their informed consent for this investigation. An immunohistochemical kit (ZSGB-BIO, Beijing, China) was used to process the staining in accordance with the instructions. Two pathologists assessed gene expression based on the ratio of tumor tissue cells and staining intensity.

### Cell lines and cell culture

Human normal lung epithelial cell line (Beas-2B; as control), H460 and 95-D cells friendly provided by Dr. Pengju Zhang. We bought HEK293T cells and NSCLC cell lines (H1299, Anip973, PC9) from Shanghai Gene Biotech in China. A549 and H358 cells were obtained from the laboratory of the Department of Pathology, Qingdao University, Qingdao, China. Each cell line was cultured according to the instructions, resuscitated at regular intervals of three months, and tested for mycoplasma.

### Plasmids, small interfering RNAs (siRNAs) and transfection

The expression plasmid Myc -USP32 (empty vector: pEnCMV-MCS-3×Myc) was purchased from Miao Ling Biotechnology Co (Wuhan, China). The expression plasmid Flag-BAG3 (empty vector: p3xFLAG-CMV-10) was purchased from GeneChem (Shanghai, China). HA-labeled Ubiquitin/HA-Tag/HA-Ubiquitin-K48/HA-Ubiquitin-K63 plasmids were previously constructed in our laboratory [36]. Control siRNA and siRNA targeting USP32 were obtained from GenePharma Company (Shanghai, China). siRNA sequences of USP32 were as follows:

USP32-siRNA-1: 5′-CGACAGUAUGGGCUAUCAATT -3′

USP32- siRNA-2: 5′-GACCUGUGGACUCUCAUAUTT -3′

Using Lipofectamine 2000 (Invitrogen) and following the manufacturer’s instructions, plasmids and siRNA were transfected into cells. 48 h after transfection, cells were collected.

### Antibodies and reagents

Purchased from Santa Cruz Biotechnology (TX, USA) were the mouse anti-USP32 antibody (sc-374465, 1:1000) and the mouse anti-MEK-1/2 antibody (sc-81504, 1:1000). The following rabbit anti-BAG3 (R23588, 1:1000), anti-E-cadherin (R22490, 1:1000), anti-p-RAF1 (R25537, 1:1000), anti-RAF1 (347271, 1:1000), anti-p-MEK1/2 (310050, 1:1000), anti-p-ERK1/2 (301245, 1:1000), and anti-ERK1/2 (R22685, 1:1000) antibodies were acquired from Zen-Bioscience (Chengdu, China). Abcam (Cambridge, MA, USA) provided rabbit anti-GAPDH (ab8245, 1:10,000) and rabbit anti-BAG3 (ab92309, 1:1000) antibodies. Cell Signaling Technologies (Danvers, MA, USA) provided the rabbit anti-Vimentin antibody (5741, 1:1000) and the mouse anti-Myc antibody (2276, 1:1000). Sigma-Aldrich (St. Louis, MO, USA) provided the mouse anti-Flag antibody (F1804, 1:1000). ZSGB-Bio Co. provided the mouse anti-HA antibody (TA-04, 1:1000) and the secondary antibody.

Calbiochem (Billerica, MA, USA) supplied the cycloheximide (CHX). Selleck PeproTech (Rocky Hill, NJ, USA) was the source of MG132. Roche (Basel, Switzerland) provided a protease inhibitor combination that was purchased. Beyotime Biotechnology (Jiangsu, China) provided the RIPA buffer. Invitrogen (Grand Island, NY, USA) provided Lipofectamine 2000. Santa Cruz (Dallas, TX, USA) supplied the protein A/G agarose.

### Western blot analysis

Utilizing protease inhibitors and protein lysis buffer RIPA (Roche, Indianapolis, IN, USA), cell lysates were produced. Bradford’s method was utilized to determine the protein concentration. Following protein samples’ separation on 10% SDS-PAGE gels, the PVDF membranes measuring 0.45 µm were placed onto them. After 5% skim milk powder in TBST was used to seal the membranes, the strips were added to the appropriate primary antibodies and incubated at 4 °C for the entire night. The directions for the dilution of the chosen antibodies were followed. The next day, the membranes were treated at room temperature for one hour with the matching enzyme-labeled secondary antibodies. The immunoreactive bands were then identified using an ECL chemiluminescence kit (GlpBio Biotechnology, USA).

### Coimmunoprecipitation

The treated cell samples were lysed with weak RIPA lysate with protease inhibitors, and centrifuged at 12,000 *g* for 15 min to obtain the lysate. A small portion of the sample was removed and boiled with 5× SDS sample buffer to be used as an Input, and the rest of the protein samples were incubated for half an hour with 10 μl of Protein A/G agarose to remove the non-specificity, and then incubated vertically overnight at 4 °C with the specific antibody added to the protein samples. The next day, 30 µl of protein A/G agarose was added to the protein samples and incubated for 4 h. After centrifugation, the agarose beads were collected and washed four times with pre-cooled IP wash solution, after which the beads were added to 2 × SDS sample buffer and boiled, and finally subjected to western blot assay.

### Deubiquitination assay

In H1299 cells, HA Ubiquitin plasmid and other required plasmids were co-transfected and treated with MG132 for 6 h and then cells were harvested and lysed with weak RIPA to extract proteins, the treated proteins were incubated with the required labeling antibodies overnight, other specifics were the same as in the Co-Immunoprecipitation experiments.

### The cycloheximide (CHX) chase and MG132 assay

Prior to the start of the experiment, CHX and MG132 were dissolved to form 50 mg/mL and 10 mM master batches, respectively, and stored at -20 °C. Cells were digested and resuspended, then spread into six-well plates and transfected by group, ensuring 2 mL of medium per well. 2 μL of CHX or MG132 was added to each well of the six-well plate, and the cells and proteins were collected at 0, 2, 4, and 8 h after CHX treatment, and at 6 h after MG132 treatment, and were detected and analyzed by western blot.

### Protein-protein docking

Rigid protein-protein docking between BAG3 and USP32 was performed using gram - x (http://gramm.compbio.ku.edu/) to study the relationship between the two. Protein structures were obtained through the Uniprot database (www.uniprot.org) as well as the Alphafold (https://alphafold.ebi.ac.uk/) database. pymol (Version 2.4) and PDBePISA (https://www.ebi.ac.uk/ pdbe/pisa/) were used for studying protein interactions and further visualization and analysis.

### Immunofluorescence microscopy

A549, H1299, and H460 cells were inoculated into 24-well plates placed in crawler sheets, and after 24 h, cell immunofluorescence was performed according to the fast and slow growth rate of cells and observation of cell density. In order to fix the cells, 0.5% Triton X-100 (PBS) was added to each well. The cells were then fixed with 4% paraformaldehyde for 20 min at room temperature, permeabilized for 20 min at room temperature with the goal of permeabilizing the cells, and blocked for two hours with 5% BSA. The blocking solution was aspirated, a sufficient amount of primary antibody of appropriate concentration was added dropwise to each well, and incubated overnight at 4 °C in a wet box. The next day, the primary antibody was removed, and the cells were treated for an hour at room temperature in the dark with a fluorescently tagged secondary antibody. Remove the crawler slice, add DAPI dropwise to the slice, and incubate for 5–10 min away from light. The absorbent paper was used to absorb the liquid on the crawler sheet, and after sealing the sheet with a sealing solution that included an anti-fluorescence quencher, it was examined under a fluorescence microscope, and pictures were taken.

### In vitro scratch/wound healing assay

Cells were digested with trypsin and resuspended in complete medium, blown well and then inoculated into 6-well plates according to the appropriate cell density, and after 24 h, scratched when the cell density reached about 90%. Scratches of roughly the same width in each well were made in six-well plates using a sterile lance tip and straightedge, after which the wells were gently rinsed with PBS and the cells were observed to photograph the 0 h scratches. Later, the state of the scratches was photographed at the corresponding desired time points.

### Transwell migration assay

The cells were starved for 12 h in a serum-free medium. After trypsin digestion, the cells were resuspended and the density was adjusted to 1 × 10^4^/ml. After activating the transwell chambers in 24-well plates, 700 μL of fetal bovine serum-containing media and 200 μL of the equivalent cell suspension were added to the lower and upper chambers, respectively. The wells were initially filled with the necessary amount of methanol solution for fixing after 48 h of incubation. This was followed by the addition of 0.1% crystal violet stain. Changes in cell migration behavior were evaluated by observing and photographing the migrating cells under a microscope.

### Cell proliferation assay

Following cell transfection, 2000 cells per well were inoculated using a cell counting technique into a 96-well plate in the CCK8(Cell Counting Kit-8) experiment, and then added CCK8 reagent according to 10 μL per well at the corresponding time point, gently mixed, and then put the 96-well plate back to the cell culture incubator to cultivate for 1-2 h, and then used the enzyme labeling instrument according to the instruction manual to detect and calculate the viability of the cells.

In clone formation experiments, cells were transfected and then inoculated in 6-well plates at about 1000 per well using cell counting. The cells were cultured for about 10 days, during which the liquid was changed every 3 days and the cell status was observed. The cells inside the wells were fixed with methanol, then the fixative was removed, 0.1% crystal violet staining was added, and finally the plates were photographed and counted.

### Statistical analysis

The statistical analysis of all the data was done with GraphPad Prism 9.5 (La Jolla, CA, USA). Every experimental result was obtained from a minimum of three separate experiments and was represented as the mean plus the standard error of the mean. One-way ANOVA or t-tests, as required for the experiment, were used to evaluate statistical significance. A statistically significant result was defined as *P* < 0.05. *p < 0.05; **p < 0.01; ***p < 0.001; ****p < 0.0001.

## Results

### USP32 is highly expressed in NSCLC and is associated with poor prognosis in NSCC patients

**Fig. 1:**
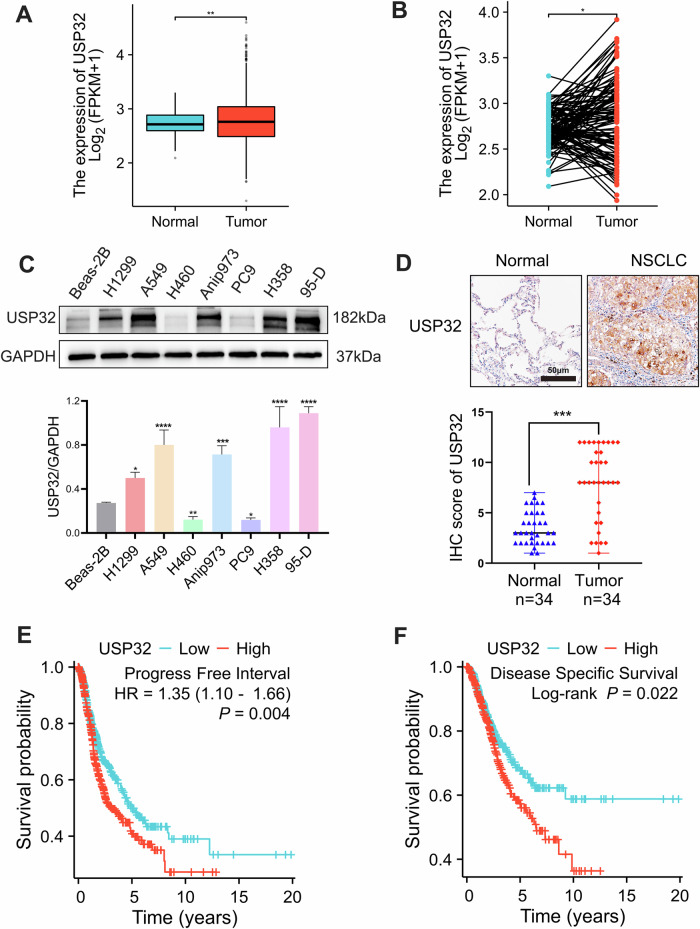
USP32 is highly expressed in NSCLC and is associated with poor prognosis in NSCLC patients. **A**, **B** Expression of USP32 mRNA in TCGA-LUAD/LUSC unpaired samples and paired paraneoplastic and carcinoma sample tissues was analyzed from the TCGA database. **C** USP32 expression in Beas-2B and 7 NSCLC cell lines by Western blot. **D** The representative immunohistochemistry staining of USP32 in NSCLC tissues and adjacent tissues (*n* = 34). Quantitative analysis was shown in the graphs. **E**, **F** TCGA database analysis of the correlation between high and low USP32 expression and NSCLC patients’ disease-free survival (DFS) and disease-specific survival (DSS). *p < 0.05; **p < 0.01; ***p < 0.001; ****p < 0.0001.

To investigate the role of USP32 in NSCLC, we first analyzed its expression level in NSCLC patients. Based on the TCGA database’s mRNA datasets for LUAD and LUSC, the analysis results from unpaired samples and paired paraneoplastic and carcinomatous samples showed that USP32 mRNA expression levels were elevated in NSCLC patients (Fig. 1A, B). For further verification, we used Western blot to identify USP32 expression in human normal lung epithelial cell lines and NSCLC cell lines. Figure 1C shows that, of the seven non-small cell lung cancer cell lines that we examined, USP 32 was up-regulated in five of them: H1299, A549, Anip973, H358, and 95-D. By employing immunohistochemistry (IHC) staining, we were able to further verify the expression of USP32 protein in NSCLC patient samples. The expression of USP32 was higher in cancer tissues compared to normal tissues adjacent to cancer (Fig. 1D). Additionally, we found that in NSCLC patients, there was a strong association between USP 32 overexpression and both disease-free survival (DFS) and disease-specific survival (DSS) (Fig. 1E, F). Together, our data suggested that USP32 may be a tumorigenic factor in non-small cell lung cancer.

### USP32 promotes NSCLC cell proliferation and migration

**Fig. 2:**
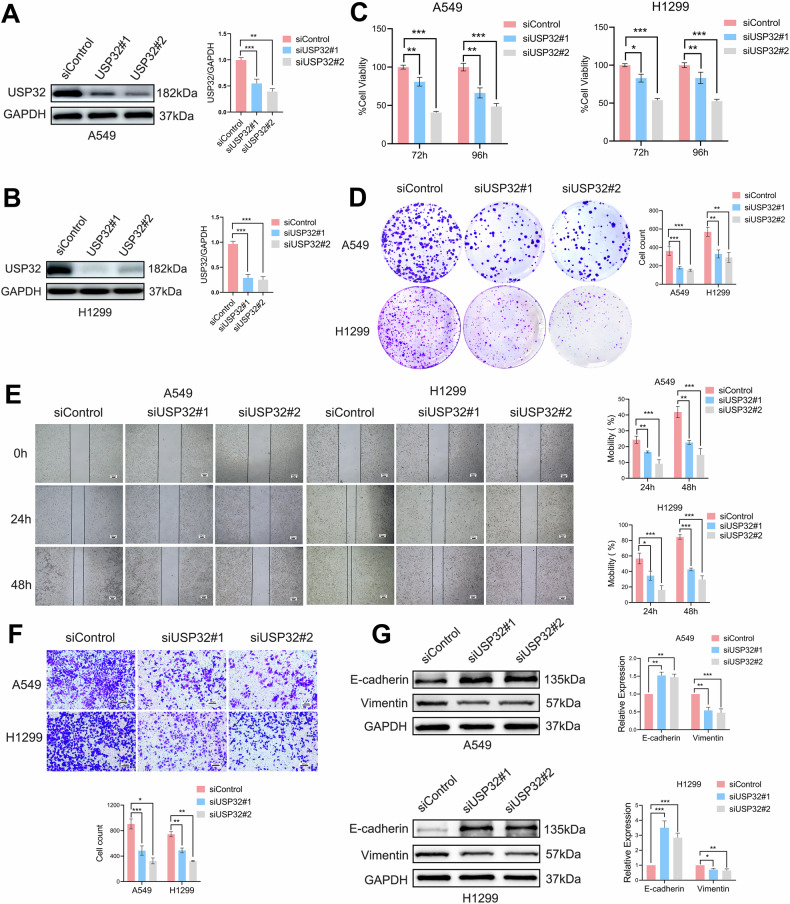
Interference with USP32 inhibits NSCLC cell proliferation and migration. **A, B** Western blot was used to detect USP32 protein level after transfection of A549/H1299 cells with siUSP32#1 and siUSP32#2. **C**, **D** CCK-8 and plate cloning assays were performed to detect change in cell proliferation ability of A549/H1299 cells transfected with siUSP32#1 and siUSP32#2 compared to control. **E**, **F** Transwell and scratch assay were used to analyze the migratory capacity of A549/H1299 cells after interference with USP32. Scale bar:100 μm. **G** The levels of epithelial-mesenchymal transition markers (E-cadherin protein, Vimentin protein) were assessed using Western blotting after interference with USP32 in A549/H1299 cells; *p < 0.05; **p < 0.01; ***p < 0.001.

The high expression of USP32 in NSCLC has been established, and we have carried out overexpression (OE) and knockdown (KD) tests on NSCLC cell lines to corroborate USP32’s biological role in NSCLC. As mentioned previously we have verified the protein expression level of USP32 by Western blot in seven selected NSCLC cell lines, we selected H460 and H1299 cells for overexpression experiment using Myc-tagged USP32 plasmid, and A549 and H1299 cells for knockdown experiment using two small interfering RNAs, siUSP32 #1, siUSP32 #2. To explore the influence of USP32 on NSCLC cell proliferation and migration, we performed a series of experiments. We first verified that USP32 was successfully knocked down in A549 and H1299 cells (Fig. 2A, B). USP32 depletion dramatically decreased the proliferation of A549 and H1299 cells, according to the results of the Cell Counting Kit-8 (CCK8) experiment (Fig. 2C). According to the clone formation assay results, A549 and H1299 single-cell clone generation were reduced when USP32 expression was knocked down in comparison to control cells (Fig. 2D). Additionally, the Transwell and Wound Healing Assay were used to evaluate migration capabilities. We discovered that USP32 knockdown decreased A549 and H1299 cells’ capacity to migrate (Fig. 2E, F). According to these findings, USP32 silencing A549 and H1299 cells exhibited increased E-cadherin and downregulated vimentin protein through Western blot analysis of molecular markers of epithelial and mesenchymal phenotypes (Fig. 2G).

USP32 overexpression plasmid was transfected into H460 and H1299 cells in order to demonstrate the effect of USP32 on NSCLC cells proliferation and migration. The plasmid’s transfection efficiency was confirmed by Western blot (Additional file 1: Fig. S1A). According to the results of CCK-8 and clone formation assay, it was obvious that cell proliferative capacity was promoted along with the upregulation of USP32 expression (Additional file 1: Fig. S1B, C). In addition, cell migration capacity was significantly enhanced in USP32 overexpressed NSCLC cells (Additional file 1: Fig. S1D, E). In the meantime, in H460 and H1299 cells, overexpression of USP32 dramatically enhanced the production of the protein vimentin and decreased the expression of the protein E-cadherin (Additional file 1: Fig. S1F, G). All of these findings demonstrate that USP32 increases the proliferation and migration process of NSCLC cells.

### USP32 interacts with BAG3 and upregulates the protein level of BAG3

**Fig. 3:**
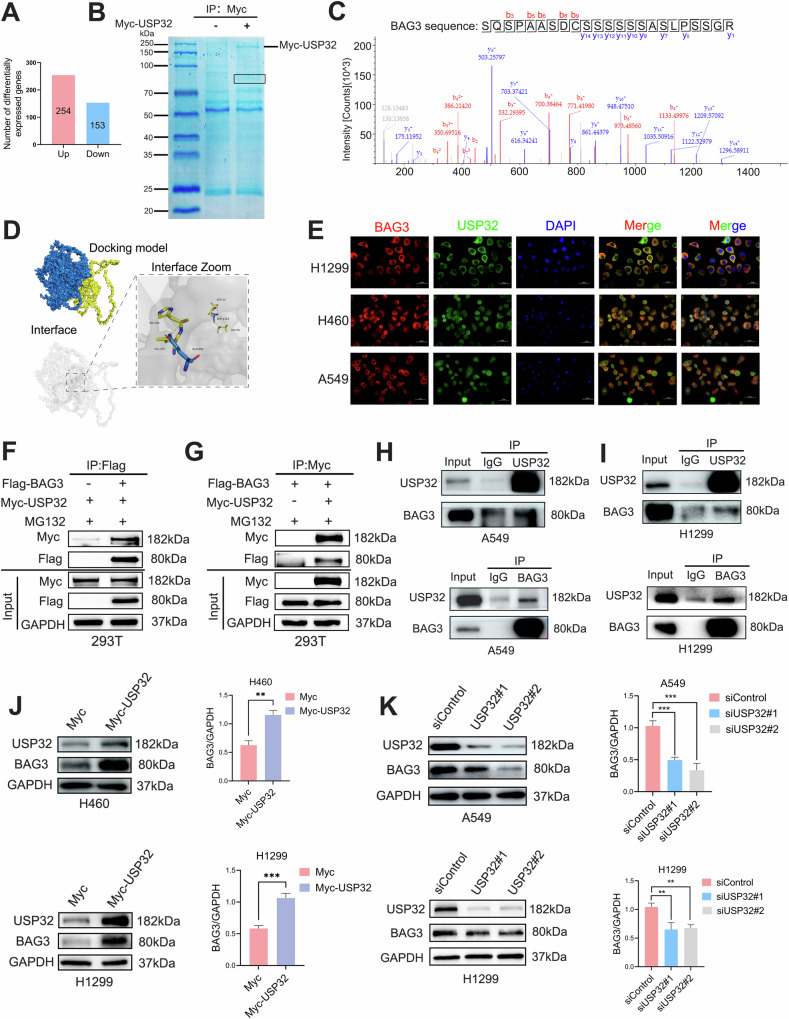
USP32 interacts with BAG3 and upregulates the protein level of BAG3. **A** The number of differentially expressed genes was screened by iTRAQ-based proteomics after overexpression of USP32 in H1299 cells. **B** H1299 cells were transiently transfected with Myc vector or Myc-USP32 expression plasmid for 48 h. Proteins were extracted from the cells and separated on a gel, which was then stained with Coomassie brilliant blue R-250. A framed protein band was evaluated by mass spectrometry analysis. **C** Representative mass spectral peaks of BAG3 interacting with Myc-USP32. **D** Docking model of USP32 and BAG3 proteins and surface map of their interfacial residues (USP32, blue; BAG3, yellow; hydrogen-bonding interactions, dotted line). **E** Co-localization studies of three cells of NSCLC using anti-USP32 antibody (1:100, green) and anti-BAG3 antibody (1:100, red) followed by DAPI nuclear counterstaining (blue). Merged images of USP32 (green) and BAG3 (red) with DAPI (blue) are also shown. Scale bar:50 μm. **F**, **G** Exogenous interaction of USP32 with BAG3. HEK293T cells were cotransfected with Myc-USP32 and/or Flag-BAG3 plasmids. Cell lysates were immunoprecipitated with the indicated antibodies and then immunoblotted with anti-Flag antibody (**F**) or anti-Myc antibody (**G**). **H**, **I** Endogenous interaction of USP32 with BAG3. A549 and H1299 cell lysates were immunoprecipitated with anti-USP32/BAG3 antibody or IgG antibody, followed by immunoblotting with anti-BAG3/USP32 antibody. **J**, **K** USP32 and BAG3 protein levels were detected by immunoblotting in USP32 overexpressed and knockdown overexpressed cells. **p < 0.01; ***p < 0.001.

Target proteins that may facilitate the promotion of USP32 for the development of non-small cell lung cancer were identified using isobaric tags for relative and absolute quantitation (iTRAQ), which was utilized to look into possible deubiquitination substrates for USP32. Out of the 6212 proteins found, 254 had an increase and 153 had a drop in USP32 overexpressed H1299 cells as compared to control cells (Fig. 3A). To further illuminate these potential target proteins regulated by USP32, we transfected H1299 cells with empty vector plasmid or Myc-tagged USP32 expression plasmid, immunoprecipitated USP32-binding proteins with anti-Myc antibody, and performed mass spectrometry analysis (Fig. 3B). Based on the results of mass spectrometry analysis, among the many potential target proteins of USP32, given that USP32 is a deubiquitinating enzyme that regulates specific roles and mechanisms of malignant tumors through deubiquitination, we paid special attention to Bcl2-associated athanogene 3 (BAG3) proteins that can be degraded by the ubiquitin protease system in this study [37] (Fig. 3C). Combining the above information, we selected BAG3 as a substrate protein for USP32 for subsequent studies. To confirm the relationship between USP32 and BAG3, we first performed protein-protein rigid docking between USP32 and BAG3, as shown in Fig. 3D and Fig. S2 (Additional file 2), BAG3 (yellow protein) formed hydrogen bonds (yellow dashed line) with USP32 (blue protein) through amino acid residue sites such as HIS-296 and GLN-952, with a binding energy of −4.0 kcal/mol, indicating that the USP32 protein formed a stable protein docking model with BAG3. This suggests a possible interaction between BAG3 (yellow protein) and USP32 (blue protein). Immunofluorescence analysis showed that in H1299, H460 and A549 cells, USP32 and BAG3 proteins were mainly co-localized in the cytoplasm (Fig. 3E). In addition, the possibility of physical contact between USP32 and BAG3 was investigated using the Co-immunoprecipitation assay (Co-IP). USP32- or BAG3-related proteins were immunoprecipitated using anti-Myc or anti-Flag antibodies, and the lysates were subjected to western blot analysis after HEK293T cells were transfected with Myc-tagged USP32 and/or Flag-tagged BAG3. USP32 interacted with BAG3, as Fig. 3F, G illustrates. Endogenous co-immunoprecipitation experiments in A549 and H1299 cells showed that USP32 could co-immunoprecipitate with BAG3 under normal medium conditions (Fig. 3H, I). These results suggest that USP32 interacts directly with BAG3.

We have demonstrated that USP32 interacts with BAG3, and further studies revealed that BAG3 protein levels were significantly elevated in USP32 overexpressing H460 and H1299 cells (Fig. 3J), whereas BAG3 protein levels were significantly reduced in USP32 knockdown A549 and H1299 cells (Fig. 3K). The above data proved that USP32 interacts with BAG3 and positively regulates BAG3 protein level.

### USP32 regulates BAG3 stability by deubiquitination function

**Fig. 4:**
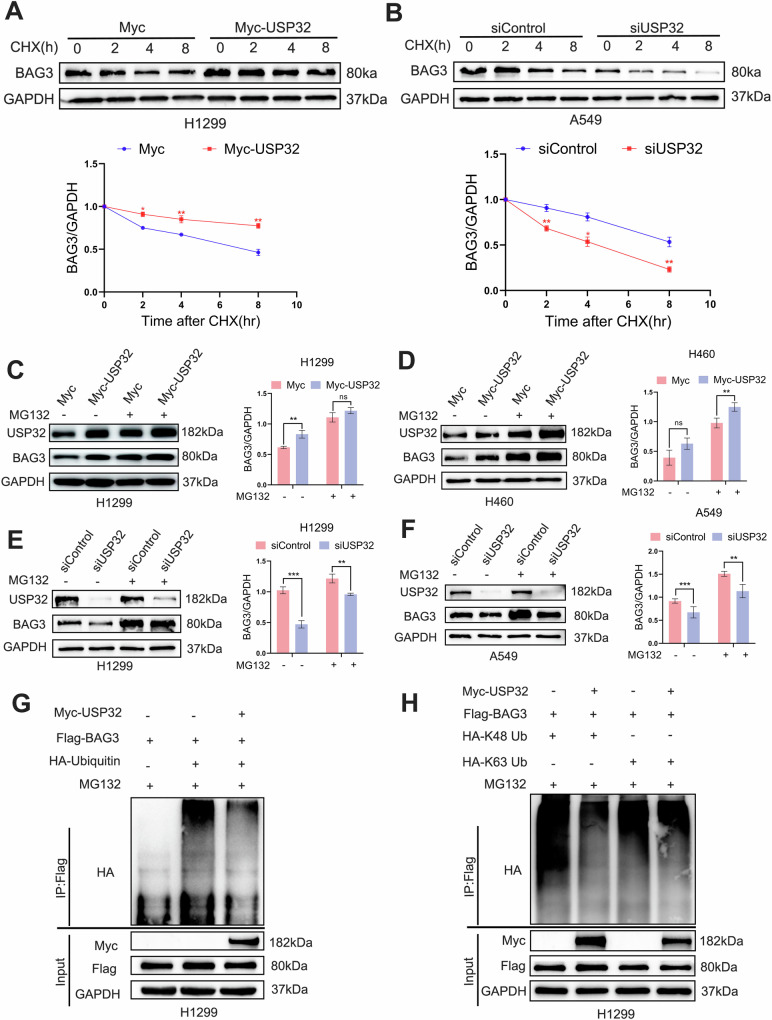
USP32 increases BAG3 stability and deubiquitinates BAG3. **A**, **B** After transfection of overexpression plasmid Myc-USP32 or small interfering siUSP32 into H1299 and A549 cells for 48 h, afterwards they were treated with 50 μg/mL cycloheximide (CHX), and the cells were collected at the indicated times, and then subjected to Western blotting analysis of BAG3 protein levels. **C**–**F** NSCLC cells after overexpression and interference with USP32 were treated with or without MG132 for 6 h, respectively, and BAG3 levels were determined by immunoblotting. **G** In the presence of Myc-vector or Myc-USP32 plasmid, HA-labeled ubiquitin and Flag-BAG3 were co-transfected with H1299 cells for 48 h, after which the cells were harvested by adding MG132 treatment for 6 h, and cell lysates were immunoprecipitated experimentally with anti-Flag antibody, and ubiquitination was detected by HA antibody. **H** H1299 cells were co-transfected with Myc- USP32/Flag- BAG3/ HA-Ubiquitin-K48/HA-Ubiquitin-K63 plasmids and treated with MG132 for 6 h. Cell lysates were immunoprecipitated with anti-Flag antibody, and ubiquitylation was detected by HA antibody. *p < 0.05; **p < 0.01; ***p < 0.001.

To investigate whether USP32 affects the stability of BAG3 protein, when USP32 siRNA or an overexpression plasmid were transfected, the BAG3 protein expression level was measured using the cycloheximide (CHX) chase assay. The findings demonstrated that USP32 overexpression in H1299 cells extended the BAG3 protein’s half-life (Fig. 4A), while USP32 knockdown accelerated the degradation of BAG3 in A549 cells (Fig. 4B). These data showed that USP32 could protect BAG3 from degradation. We then looked at whether USP32 inhibited BAG3 degradation in a way that was dependent on proteasomes. Proteasome inhibitor MG132 was used to treat H460, H1299, and A549 cells following USP32 overexpression or silencing. In all of the identified cell lines, Western blot analysis revealed that MG132 elevated the levels of BAG3 protein expression (Fig. 4C–F). These results suggested that USP32 regulated the stability of BAG3 protein through a proteasome-dependent pathway.

As a deubiquitinating enzyme, USP32 usually deubiquitinates and stabilizes substrate protein molecules. To investigate whether USP32 deubiquitinates BAG3, Myc-USP32 plasmids or a control vector were used to co-transfect Flag-BAG3 and HA-ubiquitin into H1299 cells. The cells were then treated with MG132 for six hours prior to harvesting. The degree of BAG3 ubiquitination was determined using the ubiquitin immunoblotting technique. The outcomes of the experiment demonstrated that BAG3’s degree of ubiquitination was greatly decreased by USP32 overexpression (Fig. 4G). To explore which type of ubiquitin chains on BAG3, we performed ubiquitination assays using two common types of ubiquitination (K48- and K63-linked ubiquitin chains). The findings demonstrated that whereas BAG3 ubiquitination in cells transfected with K48-linked ubiquitin chains was considerably reduced by USP32, it was unaffected in cells transfected with HA-K63-linked ubiquitin chains (Fig. 4H). To sum up, these results demonstrated that USP32 stabilizes BAG3 protein level by preventing BAG3 from ubiquitin-proteasome pathway degradation.

### BAG3 overexpression in NSCLC reverse the antitumor effect of USP32 deletion

**Fig. 5:**
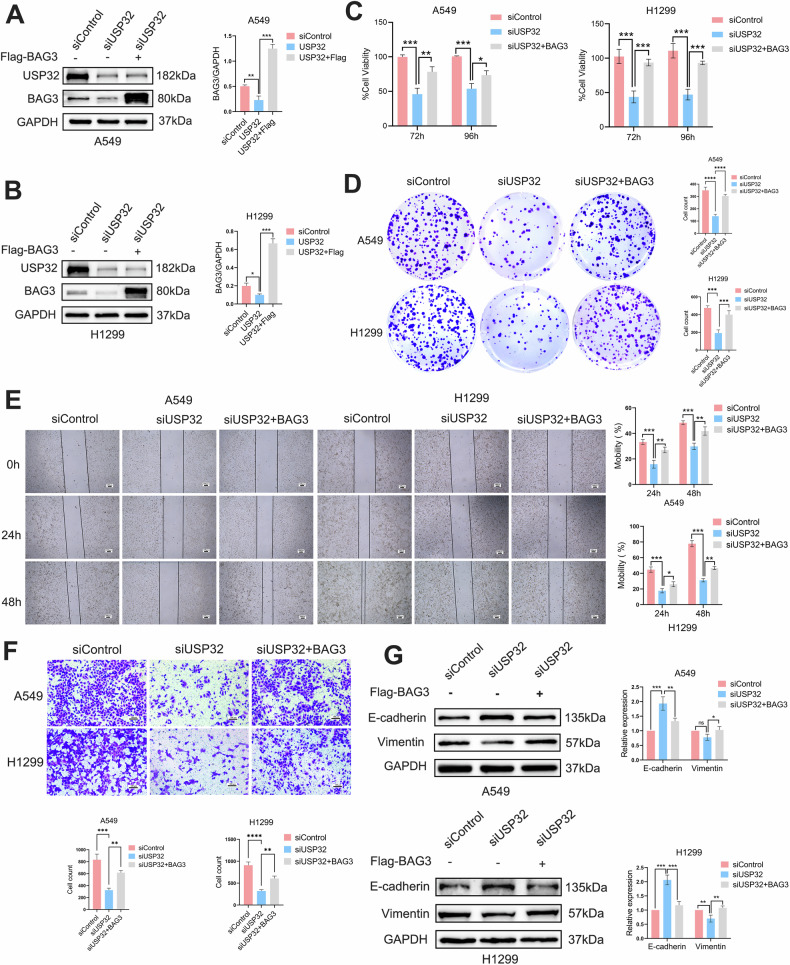
BAG3 overexpression in non-small cell lung cancer shows that USP32 deletion partially reverses antitumor effects. **A**, **B** Overexpression of BAG3 in A549 and H1299 cells after knockdown of USP32 was performed for rescue experiments, followed by detection of USP32 and BAG3 expression by Western blot. **C**–**F** BAG3 overexpression in A549 and H1299 cells restored cell proliferation and migration capacity after being knocked down by USP32. Scale bar:100 μm. **G** Changes in the expression of EMT-related proteins Vimentin and E-cadherin were detected after rescue experiments in A549 and H1299 cells. *p < 0.05; **p < 0.01; ***p < 0.001; ****p < 0.0001.

We carried out a number of rescue studies to look into the possibility that BAG3 is involved in USP32-mediated NSCLC cell proliferation, migration, and EMT. After 24 h of transfection with siControl or siUSP32, A549 and H1299 cells were transfected with Flag or Flag-BAG3. Western blot analysis demonstrated that BAG3 overexpression could reverse the knockdown of BAG3 protein level caused by siUSP32 (Fig. 5A, B). Results of CCK-8 and plate cloning experiments showed that USP32 depletion inhibited NSCLC cell lines growth and single cell clone formation, while further overexpression of BAG3 reversed NSCLC cell lines proliferation inhibition by USP32 knockdown (Fig. 5C, D). In addition, Transwell and scratch assays showed that BAG3 overexpression partially enhanced the migratory ability in the USP32 silenced NSCLC cell lines (Fig. 5E, F). In the meantime, immunoblotting research revealed that in the USP32 depleting NSCLC cell lines, E-cadherin protein was dramatically up-regulated and vimentin protein was down-regulated; however, the ectopic overexpression of BAG3 reversed the expression levels of both proteins (Fig. 5G). Based on these results, we can predict that BAG3 is an important element involved in the tumorigenic role of USP32.

### USP32 activates the RAF/MEK/ERK signaling pathway by regulating BAG3 expression

**Fig. 6:**
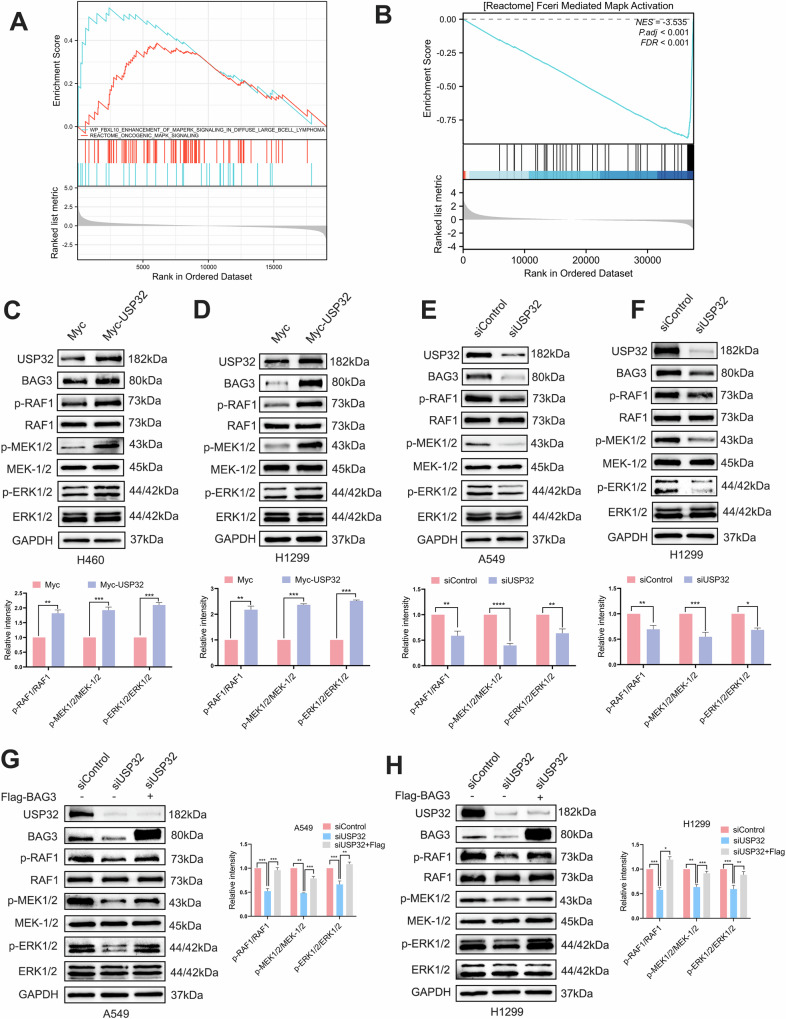
USP32 activates the RAF/MEK/ERK signaling pathway by regulating BAG3 expression. **A** GSEA enrichment of the MAPK signaling pathway in lung cancer patient-associated differential genes. Data were obtained from the TCGA database. **B** GSEA analysis shows that high expression of USP32 in lung cancer patients is associated with the MAPK signaling pathway. Data were obtained from the TCGA database. NES means normalized enrichment score. **C**, **D** Overexpression of USP32 and empty plasmid were transfected in H460 and H1299 cells, and Western blot was performed to detect the protein levels of USP32 and members of the RAF/MEK/ERK signaling pathway (RAF1, p-RAF1, MEK-1/2, p-MEK1/2, ERK1/2, and p-ERK1/2). **E**, **F** siUSP32#2 was transfected into A549 and H1299 cells, and Western blotting was performed to detect the expression of USP 32, RAF1, p-RAF1, MEK-1/2, p-MEK1/2, ERK1/2 and p-ERK1/2. **G, H** Overexpression of BAG3 activates the RAF/MEK/ERK pathway in A549 and H1299 cells of USP32 knockdown *p < 0.05; **p < 0.01; ***p < 0.001; ****p < 0.0001.

The MAPK pathway was shown to be considerably enriched by genomic enrichment analysis (GSEA) of differential genes linked with lung cancer patients in the TCGA database (Fig. 6A). Next, we used GSEA in the TCGA database to analyze the mechanism by which USP32 controls the development of NSCLC and investigate potential connections between USP32 and different signaling pathways, and as shown in Fig. 6B, MAPK_ACTIVATION was significantly enriched in NSCLCs with high levels of USP32, suggesting that the MAPK pathway is closely associated with USP32 in NSCLC. To investigate this theory, we examined the expression of key pathway molecules and discovered that, in USP32 overexpressed H460/H1299 cells, the expression of phosphorylated RAF1, phosphorylated MEK1/2, and phosphorylated ERK1/2 proteins was increased (Fig. 6C, D), indicating that the RAF/MEK/ERK signaling pathway was activated, while in A549 and H1299 cells, the expression of phosphorylated RAF1, phosphorylated MEK1/2, and phosphorylated ERK1/2 proteins was attenuated upon USP32 depletion (Fig. 6E, F). According to these findings, USP32 regulates the RAF/MEK/ERK pathway’s activation in NSCLC. We conducted rescue studies in A549 and H1299 cells by transfecting with siControl or siUSP32 for 24 h, followed by transfecting with Flag or Flag-BAG3 to examine whether USP32 activates the RAF/MEK/ERK signaling cascade by increasing BAG3 expression in NSCLC cells. Western blot analysis revealed that following BAG3 overexpression in A549 and H1299 cells with USP32 knockdown, the RAF/MEK/ERK pathway was activated (Fig. 6G, H). In summary, these findings support our theory that USP32 regulates the level of BAG3 protein to activate the RAF/MEK/ERK signaling pathway.

### In human NSCLC tissues, USP32 expression is positively correlated with BAG3 expression

**Fig. 7:**
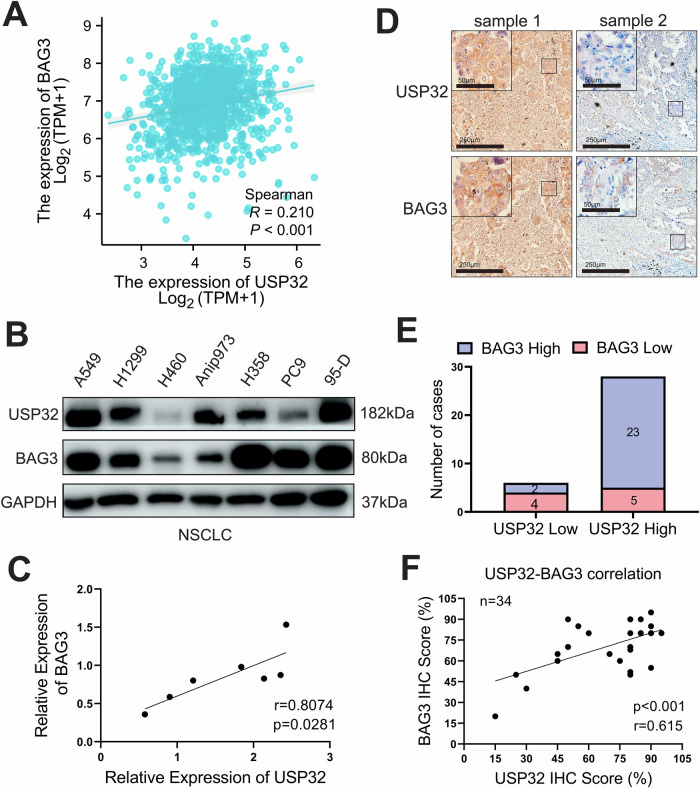
In human NSCLC tissues, USP32 expression is positively correlated with BAG3 expression. **A** Positive correlation between USP32 of LUAD/LUSC and BAG3 analyzed from the TCGA database. **B** Validation of USP32 and BAG3 expression using Western blot in multiple cell lines of NSCLC. **C** USP32 positively correlates with BAG3 in various non-small cell lung cancer cell lines. **D** Representative immunohistochemical (IHC) staining for USP32 and BAG3 in human NSCCL tissues. **E** The number of cases with negative and positive results for BAG3 and USP32 expression in NSCLC tissues were counted. **F** After 34 scoring immunostaining of NSCLC tissues, the correlation between USP32 and BAG3 expression was analyzed using scatterplots.

Given that USP32 is a DUB, we first performed an organizing and query analysis of the TCGA database to investigate the association between USP32 and BAG3. By using Spearman correlation analysis, we were able to determine that there was a positive correlation between USP32 and BAG3 in LUAD/LUSC. (Fig. 7A). After that, USP32 and BAG3 were tested in non-small cell lung cancer cell lines in order to experimentally validate their protein levels. The findings demonstrated a favorable correlation between USP32 and BAG3 expression in a variety of non-small cell lung cancer cell lines (Fig. 7B, C).

Finally, we detected and analyzed USP32-BAG3 expression and clinical correlation in human NSCLC tissues and paracancerous noncancerous tissues. We collected a total of 34 pairs of NSCLC tissues and paracancerous noncancerous tissue specimens for immunohistochemical staining, and the results were evaluated and photographed by two pathologists, which showed that USP32 and BAG3 were overexpressed in most of the NSCLC tissues, and a small number of them presented weakly positive results. The representative images are shown in Fig. 7D. Afterwards, we counted the total immunohistochemical staining, and among the 28 NSCLC tissues with high USP32 expression, there were 23 samples with elevated BAG3 expression, and among the 6 NSCLC samples with low USP32 expression, there were 4 samples with reduced BAG3 expression (Fig. 7E). Subsequently, we analyzed the relationship between USP32 and BAG3 using the percentage of positive tumor cells of USP32 and BAG3, and found that the expressions of USP32 and BAG3 in NSCLC tissues were significantly positively correlated (Fig. 7F). These data indicated that the expression of USP32 and BAG3 served as critical prognostic predictors for NSCLC patients.

## Discussion

Deubiquitinating enzymes (DUBs) are important components of the ubiquitin-proteasome system (UPS) that regulate ubiquitin signaling, thereby affecting molecular biological responses and physiological activities [38–40]. Most studies now link DUBs to cancer development and progression because dysregulation of DUBs affects the balance of the proteasome pathway, which in turn exacerbates tumorigenesis [41–43]. The drug bortezomib has been used in multiple myeloma as a proteasome inhibitor to interfere with the UPS pathway [44]. As a member of the class of deubiquitinating enzymes, USP32 has been linked to the beginnings and progression of several malignancies and has been recognized as an oncogene [11]. It has been found that USP32 is abundantly expressed in gastric cancer and that it regulates chemoresistance in GC and SMAD2 cells [45]. USP32 shows high expression in hepatocellular carcinoma and is closely associated with HCC development and immunotherapy [46]. Moreover, USP32 control over the stability of the SLC35F2 protein is linked to YM155 resistance in breast cancer [47]. The mechanism of action of USP32 in non-small cell lung cancer is currently unknown. In this work, we first analyzed the TCGA database to determine that USP32 was highly expressed in non-small cell lung cancer tissues and that this expression was correlated with a poor prognosis in NSCLC patients. We then confirmed these findings by Western blot and immunohistochemistry, which showed that USP32 was highly expressed in the majority of NSCLC cells and tissues. We carried out a number of phenotyping experiments by overexpressing and interfering with USP32 in NSCLC cells in order to better understand the biological function of USP32 in NSCLC. We discovered that USP32 expression levels significantly regulate the proliferation and migration of NSCLC tumor cells, which led us to believe that USP32 was an oncogene in NSCLC.

To clarify USP32’s mechanism of action in NSCLC, we screened proteins that may mediate USP32 to promote NSCLC development by iTRAQ proteomics analysis, and found BAG3 to be one of the up-regulated genes. Subsequently, we confirmed by protein-protein rigid docking and mass spectrometry analysis that USP32 regulates binding to BAG3. Thus, BAG3 interaction is required for USP32 to function. Because BAG3 increases the proliferation and survival of tumor cells, it is possible that BAG3 plays a significant role in the progression of tumors [48]. This result is due to the interaction between the ATPase structural domain of the HSP70 protein and the BAG structural domain of BAG3 [49]. At the same time, its WW structural domain interacts with proline-rich repeats, the PxxP structural domain interacts with the PLCγ, Src, and SH3 structural domains, and the IPV (Ile-Pro-Val) motif interacts with the small heat shock proteins, HSPB8 and HSPB631, which are involved in a wide range of life activities [50]. BAG4, another member of the BAG family, was found to bind to RAF-1, activate RAF-1 and its downstream pathway, and play an important role in promoting cell growth [51]. Furthermore, it has been shown that BAG3 is an unstable protein regulated by ubiquitination, such as FBXO22 mediates BAG3 ubiquitination and degradation during tumorigenesis [52]. However, the role of deubiquitinating enzymes in the regulation of BAG3 is unclear.

In this study, we know from immunofluorescence co-localization experiments that USP32 co-localizes with BAG3 in the cytoplasm, and that USP32 can directly bind to BAG3 by exogenous and exogenous immunoprecipitation. USP32 is overexpressed or interfered with in NSCLC cells, and the BAG3 protein is also increased or decreased. Meanwhile, in the rescue assay of overexpressing BAG3 after USP32 knockdown. It was discovered that there was a partial inhibition of NSCLC cells’ capacity to proliferate and migrate. Since USP32 is a deubiquitinating enzyme, we used ubiquitination analysis to examine the regulatory function of USP32 on BAG3. We discovered that USP32 could deubiquitinate BAG3 and that by lowering Lys48 (K48)-ubiquitination, USP32 could shield BAG3 from proteasomal degradation.

Additionally, our study’s GSEA enrichment analysis, which was based on the TCGA database, revealed that USP32 regulates the MAPK signaling pathway in NSCLC, which is implicated in both cell proliferation and apoptosis. Furthermore, this pathway’s genetic changes are frequently the cause of oncogenic mutations in human cancers [53, 54]. It has been determined that the BRAF gene is an oncogene of the RAF/MEK/ERK pathway in non-small cell lung cancer (NSCLC), leading to abnormal activation of the pathway [55, 56]. Numerous studies have demonstrated that RAF1, a key component of the MAPK signaling cascade, regulates phosphorylation and dephosphorylation in NSCLC cells in a multidirectional manner, opening up new research directions for the investigation of NSCLC pathogenesis [57, 58]. According to earlier research, ZEB1 regulation by USP10 is significantly influenced by the MEK-ERK signaling pathway [59]. Meanwhile, FBXO22 regulation of ubiquitination of BAG3 requires phosphorylation of ERK molecules [52]. Therefore, we can speculate that USP32, BAG3 and RAF/MEK/ERK pathway have some relationship. Afterwards, we activated the RAF/MEK/ERK pathway by promoting BAG3 expression through overexpression of USP32 in NSCLC and, conversely, inhibited the pathway. Remarkably, we discovered through rescue assays that BAG3 overexpression could activate the RAF/MEK/ERK pathway that siUSP32 had suppressed. Therefore, by deubiquitinating BAG3, we can show that USP32 can activate the RAF/MEK/ERK pathway.

Finally, in order to investigate the relationship between USP32-BAG3, we first found that USP32 was positively correlated with the mRNA expression of BAG3 by analyzing the TCGA database, after which their positive correlation in protein levels was verified in various cell lines of NSCLC. In addition, based on our analysis of NSCLC clinical samples, USP32 expression was also positively correlated with BAG3 protein expression. Considering these results together, we suggest that USP32 may be a positive regulator of the BAG/RAF/MEK/ERK axis in human NSCLC.

**Fig. 8:**
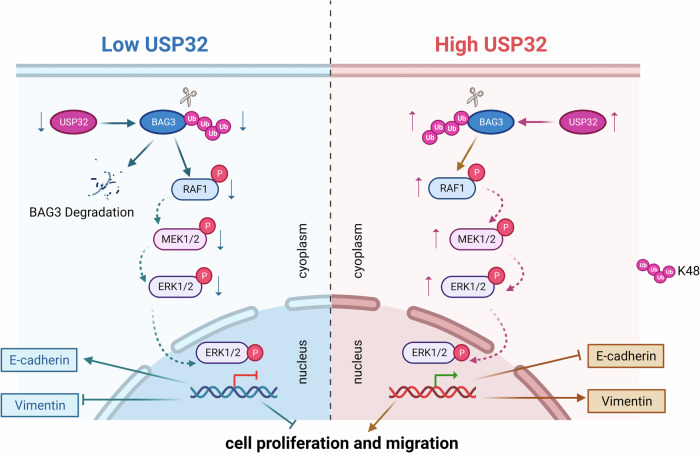
Schematic representation of the role of USP32 in NSCLC. USP32 activates the RAF/MEK/ERK signaling pathway through interaction with BAG3 and promotes the proliferation, migration and EMT of non-small cell lung cancer cells. (Figure was created with Biorender.com).

Ultimately, our study revealed that USP32 was overexpressed in NSCLC for the first time. These experimental results suggest that USP32 activates the RAF/MEK/ERK signaling pathway, which in turn promotes NSCLC cell proliferation, migration, and EMT progression through deubiquitination and stabilization of BAG3. Therefore, the USP32-BAG3-RAF/MEK/ERK axis may need to be investigated as a therapeutic target for NSCLC in the future (Fig. 8).

### Supplementary information

Supplementary Material 1

Supplementary Material 2

Supplementary Information

## Data Availability

All data associated with this study are available from the corresponding author upon request.
