# Supplementary figures and images for: USP32 facilitates non-small cell lung cancer progression via deubiquitinating BAG3 and activating RAF-MEK-ERK signaling pathway
Source: Oncogenesis. 2024 Jul 19;13(1):27. doi: 10.1038/s41389-024-00528-z (PMC11271578; doi:10.1038/s41389-024-00528-z)

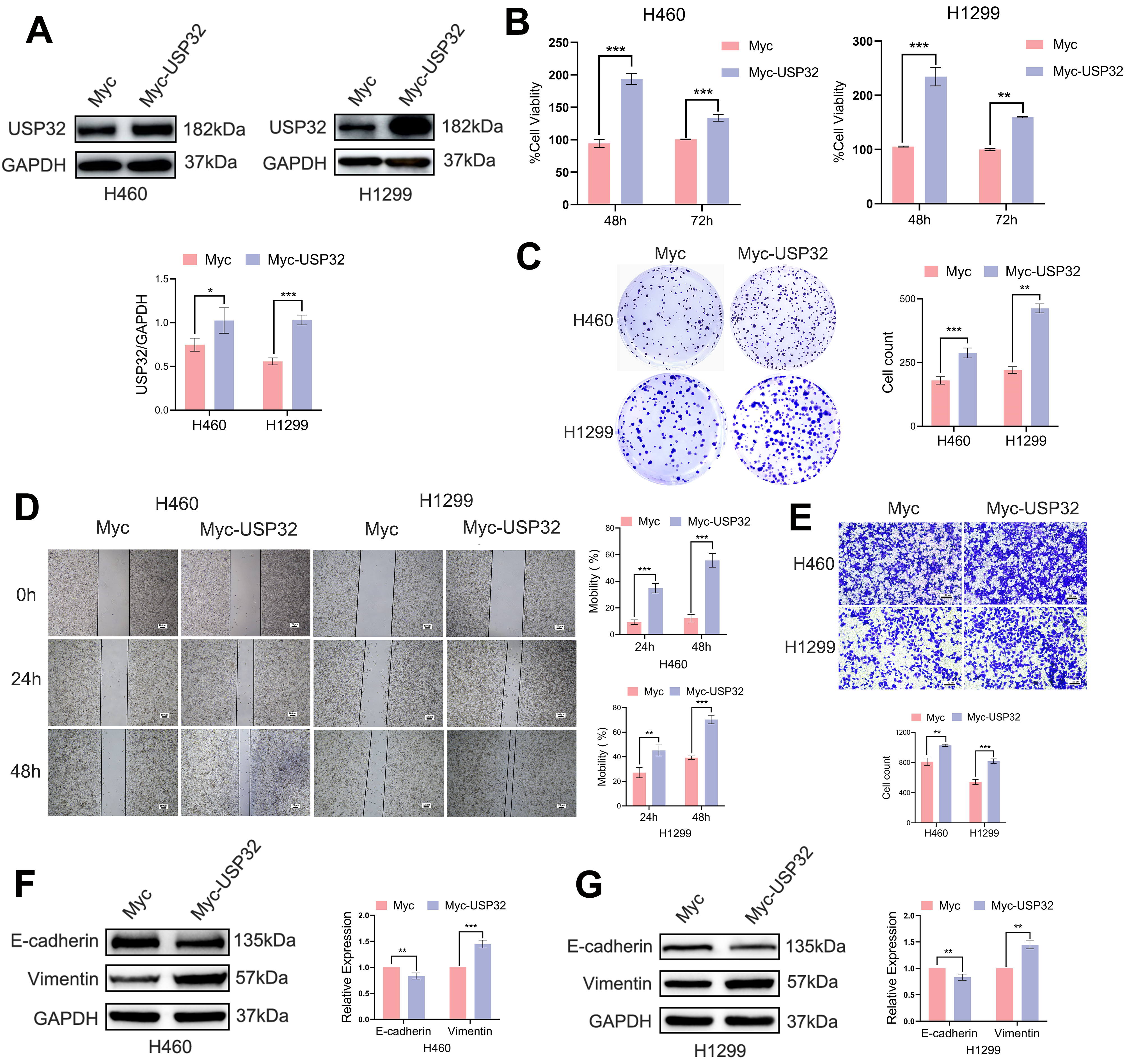

Supplement: Supplementary file 1 — Supplementary Material 1 [file 41389_2024_528_MOESM1_ESM.png]

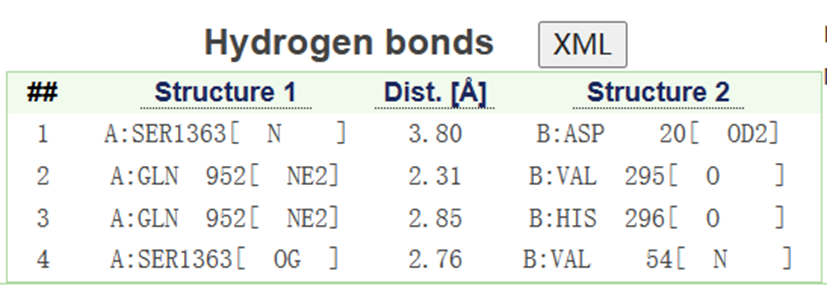

Supplement: Supplementary file 2 — Supplementary Material 2 [file 41389_2024_528_MOESM2_ESM.png]
