# Supplementary material for: USP32 facilitates non-small cell lung cancer progression via deubiquitinating BAG3 and activating RAF-MEK-ERK signaling pathway
Source: Oncogenesis. 2024 Jul 19;13(1):27. doi: 10.1038/s41389-024-00528-z (PMC11271578; doi:10.1038/s41389-024-00528-z)
Supplement: Supplementary file 3 — Supplementary Information [file 41389_2024_528_MOESM3_ESM.docx]

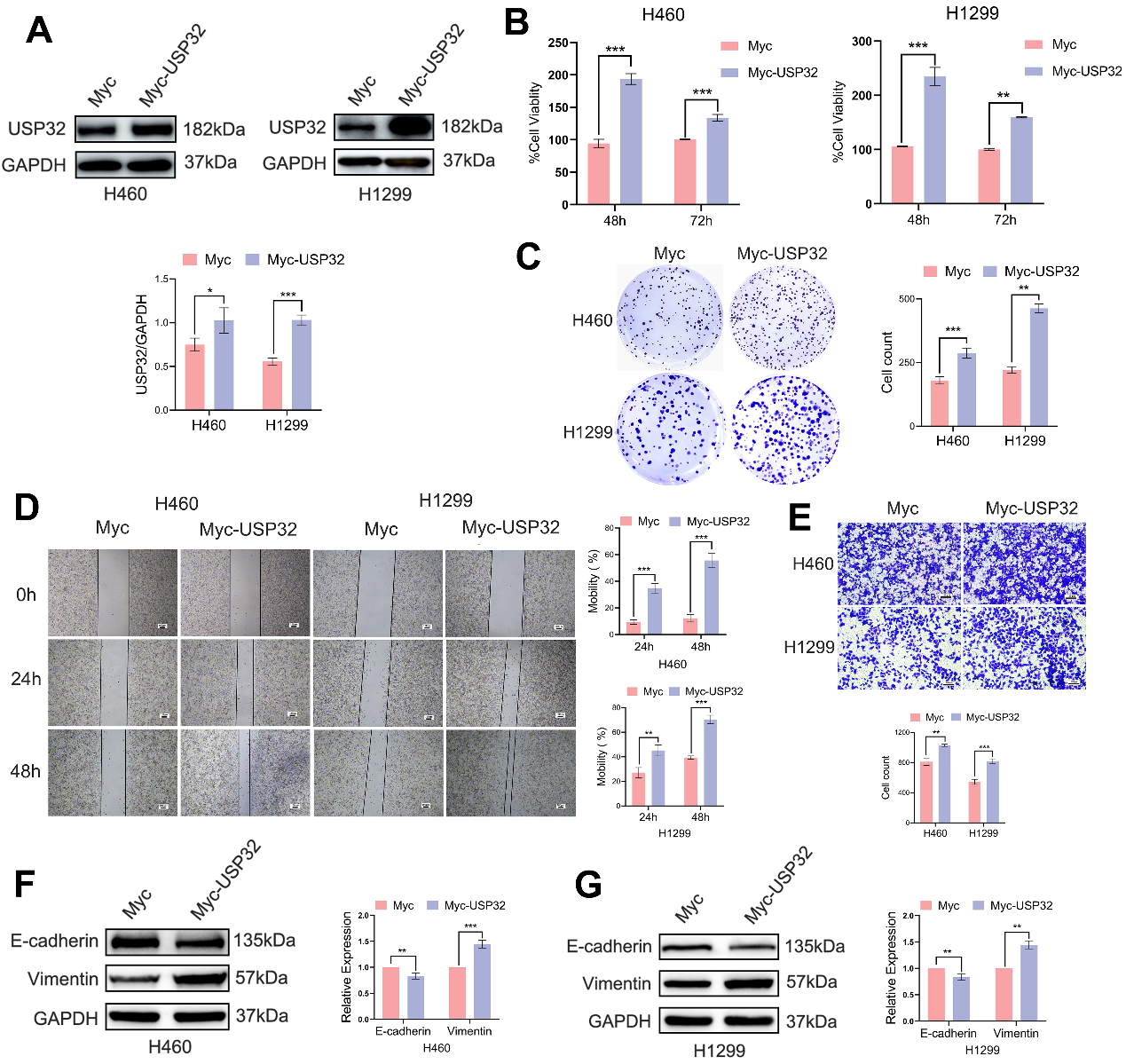
Additional file 1: **Fig. S1 Overexpression of USP32 promotes NSCLC cell proliferation and migration. (A)** USP32 overexpression plasmid and empty vector were transfected in H460/H1299 cells for 48h. Western blotting was used to detect the transfection efficiency; **(B-C)** Cell proliferative capacity of H460/H1299 cells were detected by CCK-8 and plate cloning assays after overexpression of USP32 compared to controls; **(D-E)** Transwell and scratch assays were used to analyze the migratory capacity of H460/H1299 cells after overexpression of USP32. Scale bar:100 μm; **(F-G)** Changes in levels of epithelial-mesenchymal transition markers E-cadherin and Vimentin were detected by Western blotting after transfecting with overexpression of USP32 in A549/H1299 cells. *p < 0.05; ** p < 0.01; *** p < 0.001.


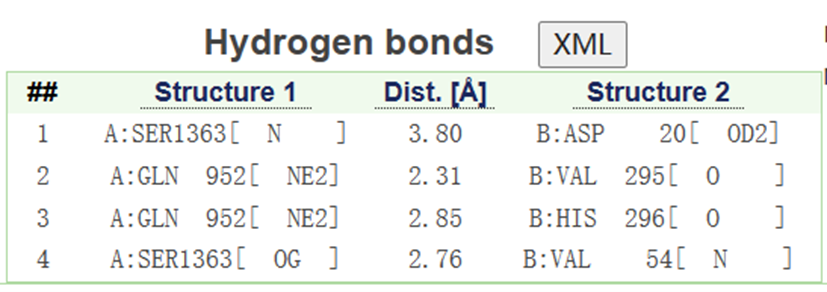


Additional file 2: Fig. S2 Amino acid residue sites formed after protein-protein rigid docking between USP32 and BAG3.
